# Crystal structure of a one-dimensional helical-type silver(I) coordination polymer: *catena*-poly[[silver(I)-μ-*N*-(pyridin-4-ylmeth­yl)pyridine-3-amine-κ^2^ *N*:*N*′] nitrate dimethyl sulfoxide disolvate]

**DOI:** 10.1107/S1600536814024817

**Published:** 2014-11-15

**Authors:** Bokhee Moon, Youngeun Jeon, Suk-Hee Moon, Ki-Min Park

**Affiliations:** a Busan International High School, Busan 614-100, Republic of Korea; b Department of Chemistry, Gyeongsang National University, Jinju 660-701, Republic of Korea; c Department of Food & Nutrition, Kyungnam College of Information and Technology, Busan 617-701, Republic of Korea; d Research Institute of Natural Sciences, Gyeongsang National University, Jinju 660-701, Republic of Korea

**Keywords:** crystal structure, silver(I) nitrate, unsymmetrical dipyridyl ligand, helical chain coordination polymer, hydrogen bonding, Ag⋯O inter­actions

## Abstract

The asymmetric unit of the title compound, {[Ag(C_11_H_11_N_3_)]NO_3_·2(CH_3_)_2_SO}_*n*_, comprises one Ag^I^ atom, one *N*-(pyridine-4-ylmeth­yl)pyridine-3-amine ligand, one nitrate anion and two dimethyl sulfoxide mol­ecules. The Ag^I^ atoms are bridged by two pyridine N atoms from two symmetry-related ligands, forming a helical chain and adopting a slightly distorted linear coordination geometry [N—Ag—N = 175.37 (8)°]. The helical chain, with a pitch length of 16.7871 (8) Å, propagates along the *b*-axis direction. In the crystal, symmetry-related right- and left-handed helical chains are alternately arranged *via* Ag⋯Ag inter­actions [3.4145 (4) Å] and π–π stacking inter­actions [centroid–centroid distance = 3.650 (2) Å], resulting in the formation of a two-dimensional supra­molecular network extending parallel to (100). Weak Ag⋯O [2.775 (2), 3.169 (4) and 2.690 (2) Å] inter­actions, as well as several N—H⋯O and C—H⋯O hydrogen-bonding inter­actions, contribute to the stabilization of the crystal structure. Parts of the dimethyl sulfoxide solvent molecule are disordered over two sets of sites in a 0.937 (3):0.063 (3) ratio.

## Abstract 2

The reaction of Ag^I^ atom with the unsymmetrical ligand *N*-(pyridin-4-ylmeth­yl)pyridine-3-amine afforded a helical chain. The Ag^I^ atom adopts a slightly distorted linear coordination geometry. The symmetry-related right- and left-handed helical chains are alternately arranged *via* Ag⋯Ag inter­actions and π–π stacking inter­actions, resulting in the formation of a two-dimensional supra­molecular network.

## Chemical context   

Self-assembled supra­molecular architectures based on the reaction of the silver ion with dipyridyl-type ligands continue to attract attention not only because of the fascinating structures caused by a variety of coordination geometries for the Ag^I^ cation, but also their potential applications as functional materials (Lee *et al.*, 2012 ▶; Leong & Vittal, 2011 ▶; Park *et al.*, 2010 ▶; Zhang *et al.*, 2009 ▶, 2013 ▶). However, although there has been rapid growth in Ag^I^ coordination chemistry based on symmetrical dipyridyl ligands with nitro­gen donor atoms in the same positions on two terminal pyridines, investigations based on unsymmetrical dipyridyl ligands with nitro­gen donor atoms in different positions on two terminal pyridines are still rare (Moon & Park, 2013 ▶, 2014 ▶; Zhang *et al.*, 2013 ▶). Therefore, the development of Ag^I^ coordination polymers using unsymmetrical dipyridyl ligands is a challenging project and deserves exploration. Herein, we report the crystal structure of the title compound prepared by the reaction of silver nitrate with the unsymmetrical dipyridyl ligand, *N*-(pyridin-4-ylmeth­yl)pyridine-3-amine, which was been synthesized by the reaction of 3-amino­pyridine and pyridine-4-carboxaldehyde according to literature methods (Foxon *et al.*, 2002 ▶; Lee *et al.*, 2013 ▶). The structure of the title compound is related to that of the monohydrated Ag^I^ coordination polymer with the same ligand (Zhang *et al.*, 2013 ▶).
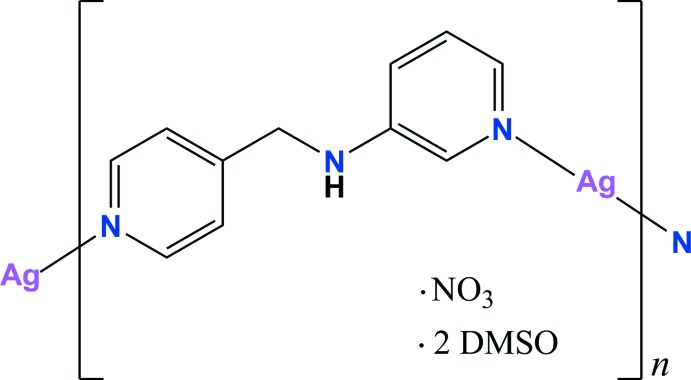


## Structural commentary   

**Figure 1:**
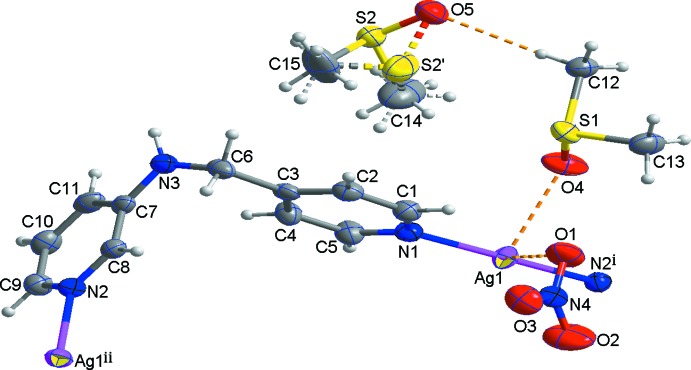
A view of the mol­ecular structure of the title compound, with the atom numbering. Displacement ellipsoids are drawn at the 50% probability level and two-coloured dashed lines indicate the disordered part of DMSO. Ag⋯O and C—H⋯O inter­actions are shown as yellow dashed lines. [Symmetry codes: (i) −*x* + 1, *y* + 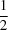, −*z* + 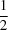; (ii) −*x* + 1, *y* − 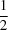, −*z* + 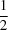.]

**Figure 2:**
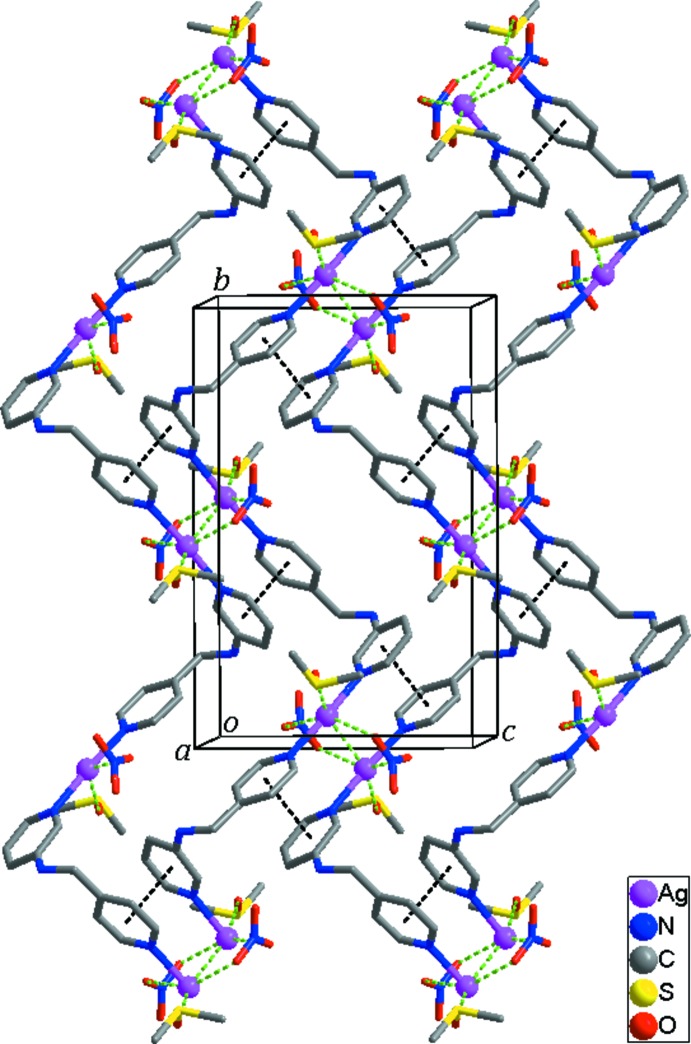
The two-dimensional supra­molecular network formed through Ag⋯Ag and Ag⋯O inter­actions (green dashed lines) as well as π–π stacking inter­actions (black dashed lines).

The mol­ecular components of the title structure are shown in Fig. 1 ▶. The asymmetric unit consists of one Ag^I^ atom, one *N*-(pyridin-4-ylmeth­yl)pyridine-3-amine ligand, one nitrate anion and two DMSO mol­ecules. The S atom of one of the DMSO mol­ecules is disordered over two sites [site-occupancy factors of 0.937 (3) for S2 and 0.063 (3) for S2′]. The Ag atom links two pyridine N atoms from two symmetry-related ligands, forming a helical chain. Thus the Ag^I^ atom is two-coordinate in a slightly distorted linear coordination geometry [N—Ag—N = 175.37 (8)°], with the Ag—N bond lengths of 2.158 (2) and 2.162 (2) Å. The helical chain propagates along the *b*-axis direction (Fig. 2 ▶) and its pitch length is 16.7871 (8) Å, much longer than that [10.135 (2) Å] of the monohydrated Ag^I^ coordination polymer reported by Zhang *et al.* (2013 ▶). The two pyridine rings coordinating to the Ag atom are tilted by 9.77 (16)° with respect to each other. In the *N*-(pyridin-4-ylmeth­yl)pyridine-3-amine ligand, the two pyridine rings are almost perpendicular, the dihedral angle between their mean planes being 86.28 (7)°.

## Supra­molecular features   

In the crystal structure, the symmetry-related right- and left-handed helical chains are alternately arranged in the structure *via* Ag⋯Ag inter­actions [3.4145 (4) Å], resulting in the formation of a two-dimensional supra­molecular network extending parallel to (100) (Fig. 2 ▶). π–π stacking inter­actions [centroid–centroid distance = 3.650 (2) Å] between the pyridine rings of both helical chains contribute to the stabilization of the two-dimensional network. The two-dimensional networks are further stabilized by Ag⋯O inter­actions [Ag1⋯O1 = 2.775 (2), Ag1⋯O2^i^ = 3.169 (4) and Ag1⋯O4 = 2.690 (2) Å; symmetry code: (i) −*x* + 1, −*y* + 1, −*z*] (Fig. 2 ▶), as well as N—H⋯O and C—H⋯O hydrogen bonds between the helical chains and the nitrate anions or the DMSO mol­ecules (Table 1 ▶). In addition, several C—H⋯O hydrogen bonds between the DMSO mol­ecules, and between the DMSO mol­ecules and the nitrate anions are also observed.

## Database survey   

The structures of the silver(I) nitrate and perchlorate complexes of the same ligand have been reported as their monohydrated and non-solvated forms, respectively, by Zhang *et al.* (2013 ▶). These complexes have been also studied for their luminescent properties in the solid state.

## Synthesis and crystallization   

*N*-(Pyridin-4-ylmeth­yl)pyridine-3-amine was prepared according to the procedure described by Lee *et al.* (2013 ▶) and Foxon *et al.* (2002 ▶). Crystals of the title compound suitable for X-ray analysis were obtained by vapour diffusion of diethyl ether into a DMSO solution of the white precipitate afforded by the reaction of the ligand with silver(I) nitrate in the molar ratio 1:1 in methanol.

## Refinement details   

Crystal data, data collection and structure refinement details are summarized in Table 2 ▶. Atoms S2 and S2′ of one DMSO mol­ecule are disordered over two sites with site-occupation factors of 0.937 (3) and 0.063 (3), respectively. All H atoms were positioned geometrically and refined using a riding model, with C—H = 0.95 Å for C*sp* ^2^—H, 0.88 Å for amine N—H and 0.99 Å for methyl­ene C—H. For all H atoms, *U*_iso_(H) = 1.2*U*_eq_(C,N).

## Supplementary Material

Crystal structure: contains datablock(s) I, New_Global_Publ_Block. DOI: 10.1107/S1600536814024817/hg5419sup1.cif

Structure factors: contains datablock(s) I. DOI: 10.1107/S1600536814024817/hg5419Isup2.hkl

CCDC reference: 1033712

Additional supporting information:  crystallographic information; 3D view; checkCIF report

## Figures and Tables

**Table 1:** Hydrogen-bond geometry (, )

*D*H*A*	*D*H	H*A*	*D* *A*	*D*H*A*
N3H3O3^i^	0.88	2.17	3.042(3)	173
C1H1O1	0.95	2.55	3.306(4)	136
C5H5O2^ii^	0.95	2.32	3.151(3)	145
C6H6*A*O3^iii^	0.99	2.42	3.405(4)	175
C8H8O3^iii^	0.95	2.55	3.480(4)	168
C10H10O4^iv^	0.95	2.44	3.309(4)	152
C12H12*A*O5	0.98	2.43	3.377(4)	161
C12H12*B*O5^v^	0.98	2.55	3.478(4)	159
C12H12*C*O3^vi^	0.98	2.35	3.270(4)	156
C13H13*A*O1	0.98	2.53	3.292(4)	134
C15H15*C*O2^i^	0.98	2.59	3.470(6)	149

Symmetry codes: (i) 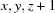; (ii) 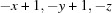; (iii) 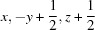; (iv) 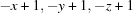; (v) 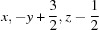; (vi) 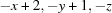.

**Table 2:** Experimental details

Crystal data	
Chemical formula	[Ag(C_11_H_11_N_3_)]NO_3_2C_2_H_6_OS
*M*_r_	511.36
Crystal system, space group	Monoclinic, *P*2_1_/*c*
Temperature (K)	173
*a*, *b*, *c* ()	11.7046(6), 16.7871(8), 10.4922(5)
()	91.950(1)
*V* (^3^)	2060.38(17)
*Z*	4
Radiation type	Mo *K*
(mm^1^)	1.21
Crystal size (mm)	0.31 0.24 0.12

Data collection	
Diffractometer	Bruker SMART CCD
Absorption correction	Multi-scan (*SADABS*; Bruker, 2000 ▶)
*T*_min_, *T*_max_	0.705, 0.868
No. of measured, independent and observed [*I* > 2(*I*)] reflections	11476, 4039, 3485
*R*_int_	0.063
(sin /)_max_ (^1^)	0.617

Refinement	
*R*[*F* ^2^ > 2(*F* ^2^)], *wR*(*F* ^2^), *S*	0.028, 0.078, 1.08
No. of reflections	4039
No. of parameters	255
H-atom treatment	H-atom parameters constrained
_max_, _min_ (e ^3^)	0.58, 0.63

Computer programs: *SMART* and *SAINT-Plus* (Bruker, 2000 ▶), *SHELXS97*, *SHELXL97* and *SHELXTL* (Sheldrick, 2008 ▶) and *DIAMOND* (Brandenburg, 2005 ▶).

## supplementary crystallographic information

### Crystal data

**Table:**

[Ag(C_11_H_11_N_3_)]NO_3_·2C_2_H_6_OS	*F*(000) = 1040
*M**_r_* = 511.36	*D*_x_ = 1.649 Mg m^−^^3^
Monoclinic, *P*2_1_/*c*	Mo *K*α radiation, λ = 0.71073 Å
Hall symbol: -P 2ybc	Cell parameters from 7555 reflections
*a* = 11.7046 (6) Å	θ = 2.3–28.3°
*b* = 16.7871 (8) Å	µ = 1.21 mm^−^^1^
*c* = 10.4922 (5) Å	*T* = 173 K
β = 91.950 (1)°	Plate, colorless
*V* = 2060.38 (17) Å^3^	0.31 × 0.24 × 0.12 mm
*Z* = 4	

### Data collection

**Table:**

Bruker SMART CCD area-detector diffractometer	4039 independent reflections
Radiation source: fine-focus sealed tube	3485 reflections with *I* > 2σ(*I*)
Graphite monochromator	*R*_int_ = 0.063
φ and ω scans	θ_max_ = 26.0°, θ_min_ = 1.7°
Absorption correction: multi-scan (*SADABS*; Bruker, 2000)	*h* = −12→14
*T*_min_ = 0.705, *T*_max_ = 0.868	*k* = −20→19
11476 measured reflections	*l* = −12→12

### Refinement

**Table:**

Refinement on *F*^2^	Primary atom site location: structure-invariant direct methods
Least-squares matrix: full	Secondary atom site location: difference Fourier map
*R*[*F*^2^ > 2σ(*F*^2^)] = 0.028	Hydrogen site location: inferred from neighbouring sites
*wR*(*F*^2^) = 0.078	H-atom parameters constrained
*S* = 1.08	*w* = 1/[σ^2^(*F*_o_^2^) + (0.0348*P*)^2^ + 1.0473*P*] where *P* = (*F*_o_^2^ + 2*F*_c_^2^)/3
4039 reflections	(Δ/σ)_max_ = 0.001
255 parameters	Δρ_max_ = 0.58 e Å^−^^3^
0 restraints	Δρ_min_ = −0.63 e Å^−^^3^

### Special details

**Table:**

Geometry. All e.s.d.'s (except the e.s.d. in the dihedral angle between two l.s. planes) are estimated using the full covariance matrix. The cell e.s.d.'s are taken into account individually in the estimation of e.s.d.'s in distances, angles and torsion angles; correlations between e.s.d.'s in cell parameters are only used when they are defined by crystal symmetry. An approximate (isotropic) treatment of cell e.s.d.'s is used for estimating e.s.d.'s involving l.s. planes.
Refinement. Refinement of *F*^2^ against ALL reflections. The weighted *R*-factor *wR* and goodness of fit *S* are based on *F*^2^, conventional *R*-factors *R* are based on *F*, with *F* set to zero for negative *F*^2^. The threshold expression of *F*^2^ > σ(*F*^2^) is used only for calculating *R*-factors(gt) *etc*. and is not relevant to the choice of reflections for refinement. *R*-factors based on *F*^2^ are statistically about twice as large as those based on *F*, and *R*- factors based on ALL data will be even larger.

### Fractional atomic coordinates and isotropic or equivalent isotropic displacement parameters (Å^2^)

**Table:**

	*x*	*y*	*z*	*U*_iso_*/*U*_eq_	Occ. (<1)
Ag1	0.599664 (17)	0.557957 (11)	0.078155 (18)	0.03159 (9)	
N1	0.64574 (18)	0.46711 (13)	0.2167 (2)	0.0311 (5)	
N2	0.45491 (18)	0.15285 (12)	0.5486 (2)	0.0287 (5)	
N3	0.6908 (2)	0.29282 (13)	0.6162 (2)	0.0346 (5)	
H3	0.7182	0.3160	0.6859	0.092 (15)*	
C1	0.7468 (2)	0.42880 (15)	0.2103 (3)	0.0328 (6)	
H1	0.7945	0.4404	0.1412	0.039*	
C2	0.7836 (2)	0.37356 (15)	0.2997 (3)	0.0321 (6)	
H2	0.8551	0.3477	0.2911	0.039*	
C3	0.7164 (2)	0.35560 (14)	0.4024 (2)	0.0279 (5)	
C4	0.6126 (2)	0.39512 (16)	0.4087 (3)	0.0355 (6)	
H4	0.5635	0.3849	0.4772	0.043*	
C5	0.5808 (2)	0.44944 (16)	0.3151 (3)	0.0373 (6)	
H5	0.5092	0.4755	0.3211	0.045*	
C6	0.7578 (2)	0.29614 (15)	0.5034 (3)	0.0336 (6)	
H6A	0.7585	0.2425	0.4644	0.040*	
H6B	0.8375	0.3096	0.5294	0.040*	
C7	0.5858 (2)	0.25525 (14)	0.6209 (2)	0.0284 (5)	
C8	0.5553 (2)	0.19125 (14)	0.5408 (2)	0.0270 (5)	
H8	0.6074	0.1744	0.4788	0.032*	
C9	0.3803 (2)	0.17604 (17)	0.6358 (3)	0.0350 (6)	
H9	0.3100	0.1483	0.6420	0.042*	
C10	0.4037 (3)	0.23941 (17)	0.7165 (3)	0.0388 (6)	
H10	0.3495	0.2553	0.7769	0.047*	
C11	0.5062 (2)	0.27946 (16)	0.7091 (2)	0.0356 (6)	
H11	0.5226	0.3234	0.7639	0.043*	
S1	0.90399 (6)	0.61772 (4)	0.14218 (7)	0.03541 (16)	
O4	0.78359 (17)	0.64866 (14)	0.1364 (2)	0.0545 (6)	
C12	0.9849 (2)	0.68750 (17)	0.2364 (3)	0.0392 (7)	
H12A	0.9627	0.6838	0.3254	0.047*	
H12B	0.9699	0.7415	0.2046	0.047*	
H12C	1.0665	0.6755	0.2309	0.047*	
C13	0.9641 (3)	0.6403 (2)	−0.0077 (3)	0.0470 (7)	
H13A	0.9288	0.6065	−0.0741	0.071*	
H13B	1.0467	0.6305	−0.0025	0.071*	
H13C	0.9500	0.6964	−0.0287	0.071*	
S2	0.85523 (7)	0.61054 (5)	0.61168 (7)	0.0404 (3)	0.937 (3)
S2'	0.8683 (14)	0.5787 (10)	0.4964 (13)	0.060 (5)	0.063 (3)
O5	0.95718 (18)	0.64284 (13)	0.5476 (2)	0.0486 (5)	
C14	0.7356 (3)	0.6311 (3)	0.5101 (4)	0.0866 (14)	
H14A	0.7204	0.6885	0.5099	0.104*	0.937 (3)
H14B	0.6687	0.6026	0.5407	0.104*	0.937 (3)
H14C	0.7512	0.6135	0.4234	0.104*	0.937 (3)
H14D	0.7343	0.6771	0.4527	0.104*	0.063 (3)
H14E	0.7282	0.6493	0.5982	0.104*	0.063 (3)
H14F	0.6719	0.5954	0.4870	0.104*	0.063 (3)
C15	0.8616 (4)	0.5055 (2)	0.5930 (5)	0.0829 (14)	
H15A	0.9245	0.4841	0.6466	0.100*	0.937 (3)
H15B	0.8743	0.4926	0.5035	0.100*	0.937 (3)
H15C	0.7893	0.4818	0.6185	0.100*	0.937 (3)
H15D	0.9322	0.4742	0.5905	0.100*	0.063 (3)
H15E	0.7963	0.4716	0.5681	0.100*	0.063 (3)
H15F	0.8519	0.5256	0.6796	0.100*	0.063 (3)
N4	0.72721 (19)	0.45059 (12)	−0.1442 (2)	0.0309 (5)	
O1	0.7696 (2)	0.50433 (13)	−0.0788 (2)	0.0544 (6)	
O2	0.6392 (2)	0.46146 (17)	−0.2082 (3)	0.0807 (10)	
O3	0.77059 (19)	0.38352 (12)	−0.1461 (2)	0.0521 (6)	

### Atomic displacement parameters (Å^2^)

**Table:**

	*U*^11^	*U*^22^	*U*^33^	*U*^12^	*U*^13^	*U*^23^
Ag1	0.03243 (13)	0.03166 (13)	0.03064 (12)	0.00682 (8)	0.00031 (9)	0.00448 (8)
N1	0.0263 (11)	0.0307 (11)	0.0361 (12)	0.0013 (9)	−0.0021 (9)	0.0028 (9)
N2	0.0270 (11)	0.0292 (11)	0.0298 (11)	−0.0012 (8)	0.0004 (9)	−0.0015 (9)
N3	0.0403 (13)	0.0306 (11)	0.0324 (12)	−0.0062 (9)	−0.0075 (10)	0.0020 (9)
C1	0.0271 (13)	0.0353 (14)	0.0360 (14)	0.0018 (10)	0.0035 (11)	−0.0005 (11)
C2	0.0249 (12)	0.0312 (13)	0.0402 (14)	0.0039 (10)	−0.0003 (11)	−0.0028 (11)
C3	0.0261 (12)	0.0207 (11)	0.0365 (13)	−0.0034 (9)	−0.0055 (11)	−0.0014 (10)
C4	0.0284 (13)	0.0354 (14)	0.0427 (15)	0.0014 (11)	0.0040 (12)	0.0086 (12)
C5	0.0266 (13)	0.0375 (15)	0.0480 (17)	0.0055 (11)	0.0022 (12)	0.0085 (12)
C6	0.0279 (13)	0.0272 (13)	0.0451 (15)	−0.0028 (10)	−0.0075 (12)	0.0051 (11)
C7	0.0359 (14)	0.0239 (12)	0.0249 (11)	−0.0003 (10)	−0.0053 (10)	0.0037 (10)
C8	0.0285 (12)	0.0272 (12)	0.0253 (12)	0.0015 (10)	−0.0010 (10)	−0.0009 (9)
C9	0.0301 (13)	0.0391 (15)	0.0358 (14)	0.0001 (11)	0.0030 (11)	−0.0003 (12)
C10	0.0436 (16)	0.0423 (16)	0.0309 (13)	0.0084 (13)	0.0071 (12)	−0.0037 (12)
C11	0.0474 (16)	0.0306 (13)	0.0283 (12)	0.0044 (12)	−0.0044 (12)	−0.0053 (11)
S1	0.0319 (3)	0.0323 (3)	0.0423 (4)	−0.0020 (3)	0.0062 (3)	−0.0029 (3)
O4	0.0266 (10)	0.0654 (14)	0.0718 (15)	−0.0028 (10)	0.0073 (10)	−0.0272 (13)
C12	0.0343 (15)	0.0469 (17)	0.0360 (14)	−0.0019 (12)	−0.0018 (12)	−0.0044 (13)
C13	0.0429 (17)	0.063 (2)	0.0353 (15)	−0.0057 (15)	0.0019 (13)	−0.0093 (14)
S2	0.0469 (5)	0.0423 (5)	0.0318 (4)	−0.0074 (3)	0.0008 (3)	−0.0013 (3)
S2'	0.057 (9)	0.081 (11)	0.040 (7)	−0.001 (7)	0.004 (6)	0.001 (7)
O5	0.0421 (12)	0.0470 (12)	0.0565 (13)	−0.0080 (9)	0.0009 (10)	0.0018 (10)
C14	0.043 (2)	0.141 (4)	0.075 (3)	−0.001 (2)	−0.001 (2)	0.009 (3)
C15	0.113 (4)	0.042 (2)	0.095 (3)	−0.023 (2)	0.024 (3)	−0.003 (2)
N4	0.0269 (11)	0.0321 (12)	0.0340 (12)	0.0015 (9)	0.0039 (9)	−0.0025 (9)
O1	0.0673 (15)	0.0464 (12)	0.0498 (13)	−0.0149 (11)	0.0055 (11)	−0.0193 (11)
O2	0.0514 (15)	0.0690 (17)	0.119 (3)	0.0119 (13)	−0.0377 (17)	0.0006 (17)
O3	0.0473 (13)	0.0342 (11)	0.0748 (16)	0.0130 (9)	0.0028 (12)	0.0001 (11)

### Geometric parameters (Å, º)

**Table:**

Ag1—N2^i^	2.158 (2)	S1—C13	1.785 (3)
Ag1—N1	2.162 (2)	C12—H12A	0.9800
N1—C5	1.337 (4)	C12—H12B	0.9800
N1—C1	1.350 (3)	C12—H12C	0.9800
N2—C9	1.343 (4)	C13—H13A	0.9800
N2—C8	1.345 (3)	C13—H13B	0.9800
N2—Ag1^ii^	2.158 (2)	C13—H13C	0.9800
N3—C7	1.383 (3)	S2—O5	1.492 (2)
N3—C6	1.443 (4)	S2—C14	1.764 (4)
N3—H3	0.8800	S2—C15	1.777 (4)
C1—C2	1.378 (4)	S2—H14E	1.6245
C1—H1	0.9500	S2—H15F	1.5946
C2—C3	1.389 (4)	S2'—O5	1.579 (16)
C2—H2	0.9500	S2'—C15	1.597 (16)
C3—C4	1.388 (4)	S2'—C14	1.794 (17)
C3—C6	1.523 (3)	C14—H14A	0.9800
C4—C5	1.382 (4)	C14—H14B	0.9800
C4—H4	0.9500	C14—H14C	0.9800
C5—H5	0.9500	C14—H14D	0.9799
C6—H6A	0.9900	C14—H14E	0.9799
C6—H6B	0.9900	C14—H14F	0.9800
C7—C11	1.396 (4)	C15—H15A	0.9800
C7—C8	1.402 (3)	C15—H15B	0.9800
C8—H8	0.9500	C15—H15C	0.9800
C9—C10	1.381 (4)	C15—H15D	0.9800
C9—H9	0.9500	C15—H15E	0.9801
C10—C11	1.380 (4)	C15—H15F	0.9799
C10—H10	0.9500	N4—O2	1.224 (3)
C11—H11	0.9500	N4—O1	1.228 (3)
S1—O4	1.501 (2)	N4—O3	1.236 (3)
S1—C12	1.784 (3)		
			
N2^i^—Ag1—N1	175.37 (8)	O5—S2—H14E	124.0
C5—N1—C1	117.0 (2)	C15—S2—H14E	115.4
C5—N1—Ag1	122.74 (17)	O5—S2—H15F	124.0
C1—N1—Ag1	120.11 (19)	C14—S2—H15F	114.4
C9—N2—C8	119.5 (2)	H14E—S2—H15F	111.1
C9—N2—Ag1^ii^	116.62 (17)	O5—S2'—C15	110.8 (9)
C8—N2—Ag1^ii^	123.85 (17)	O5—S2'—C14	101.6 (9)
C7—N3—C6	123.7 (2)	C15—S2'—C14	105.3 (9)
C7—N3—H3	118.2	S2—O5—S2'	51.5 (6)
C6—N3—H3	118.2	S2—C14—H14A	109.5
N1—C1—C2	122.7 (3)	S2'—C14—H14A	129.7
N1—C1—H1	118.6	S2—C14—H14B	109.5
C2—C1—H1	118.6	S2'—C14—H14B	119.4
C1—C2—C3	120.2 (2)	H14A—C14—H14B	109.5
C1—C2—H2	119.9	S2—C14—H14C	109.5
C3—C2—H2	119.9	S2'—C14—H14C	65.7
C4—C3—C2	116.9 (2)	H14A—C14—H14C	109.5
C4—C3—C6	122.6 (2)	H14B—C14—H14C	109.5
C2—C3—C6	120.5 (2)	S2—C14—H14D	121.4
C5—C4—C3	119.8 (3)	S2'—C14—H14D	109.4
C5—C4—H4	120.1	H14B—C14—H14D	126.1
C3—C4—H4	120.1	H14C—C14—H14D	70.5
N1—C5—C4	123.3 (3)	S2—C14—H14E	65.5
N1—C5—H5	118.3	S2'—C14—H14E	109.5
C4—C5—H5	118.3	H14A—C14—H14E	70.9
N3—C6—C3	115.3 (2)	H14B—C14—H14E	75.3
N3—C6—H6A	108.5	H14C—C14—H14E	174.3
C3—C6—H6A	108.5	H14D—C14—H14E	109.5
N3—C6—H6B	108.5	S2—C14—H14F	127.7
C3—C6—H6B	108.5	S2'—C14—H14F	109.5
H6A—C6—H6B	107.5	H14A—C14—H14F	117.6
N3—C7—C11	120.3 (2)	H14C—C14—H14F	75.5
N3—C7—C8	122.5 (2)	H14D—C14—H14F	109.5
C11—C7—C8	117.2 (2)	H14E—C14—H14F	109.5
N2—C8—C7	122.4 (2)	S2'—C15—S2	46.3 (6)
N2—C8—H8	118.8	S2'—C15—H15A	126.5
C7—C8—H8	118.8	S2—C15—H15A	109.5
N2—C9—C10	121.4 (3)	S2'—C15—H15B	63.2
N2—C9—H9	119.3	S2—C15—H15B	109.5
C10—C9—H9	119.3	H15A—C15—H15B	109.5
C11—C10—C9	119.7 (3)	S2'—C15—H15C	123.2
C11—C10—H10	120.2	S2—C15—H15C	109.5
C9—C10—H10	120.2	H15A—C15—H15C	109.5
C10—C11—C7	119.8 (2)	H15B—C15—H15C	109.5
C10—C11—H11	120.1	S2'—C15—H15D	109.6
C7—C11—H11	120.1	S2—C15—H15D	124.9
O4—S1—C12	105.96 (13)	H15B—C15—H15D	72.5
O4—S1—C13	106.77 (15)	H15C—C15—H15D	121.8
C12—S1—C13	97.50 (14)	S2'—C15—H15E	109.4
S1—C12—H12A	109.5	S2—C15—H15E	124.8
S1—C12—H12B	109.5	H15A—C15—H15E	120.3
H12A—C12—H12B	109.5	H15B—C15—H15E	76.1
S1—C12—H12C	109.5	H15D—C15—H15E	109.5
H12A—C12—H12C	109.5	S2'—C15—H15F	109.4
H12B—C12—H12C	109.5	S2—C15—H15F	63.1
S1—C13—H13A	109.5	H15A—C15—H15F	72.7
S1—C13—H13B	109.5	H15B—C15—H15F	172.3
H13A—C13—H13B	109.5	H15C—C15—H15F	76.1
S1—C13—H13C	109.5	H15D—C15—H15F	109.5
H13A—C13—H13C	109.5	H15E—C15—H15F	109.5
H13B—C13—H13C	109.5	O2—N4—O1	120.9 (2)
O5—S2—C14	106.70 (18)	O2—N4—O3	117.7 (2)
O5—S2—C15	105.90 (18)	O1—N4—O3	121.3 (2)
C14—S2—C15	99.3 (3)		
			
C5—N1—C1—C2	0.1 (4)	C8—N2—C9—C10	1.0 (4)
Ag1—N1—C1—C2	176.35 (19)	Ag1^ii^—N2—C9—C10	−176.0 (2)
N1—C1—C2—C3	−0.5 (4)	N2—C9—C10—C11	−0.8 (4)
C1—C2—C3—C4	0.4 (4)	C9—C10—C11—C7	−0.5 (4)
C1—C2—C3—C6	−178.5 (2)	N3—C7—C11—C10	−177.3 (2)
C2—C3—C4—C5	0.0 (4)	C8—C7—C11—C10	1.5 (4)
C6—C3—C4—C5	178.9 (2)	C14—S2—O5—S2'	−58.3 (8)
C1—N1—C5—C4	0.3 (4)	C15—S2—O5—S2'	46.9 (8)
Ag1—N1—C5—C4	−175.8 (2)	C15—S2'—O5—S2	−56.6 (7)
C3—C4—C5—N1	−0.4 (4)	C14—S2'—O5—S2	54.9 (5)
C7—N3—C6—C3	76.5 (3)	O5—S2—C14—S2'	57.3 (8)
C4—C3—C6—N3	−9.4 (3)	C15—S2—C14—S2'	−52.5 (8)
C2—C3—C6—N3	169.5 (2)	O5—S2'—C14—S2	−51.1 (5)
C6—N3—C7—C11	−154.1 (2)	C15—S2'—C14—S2	64.6 (6)
C6—N3—C7—C8	27.1 (4)	O5—S2'—C15—S2	49.4 (6)
C9—N2—C8—C7	0.0 (4)	C14—S2'—C15—S2	−59.7 (7)
Ag1^ii^—N2—C8—C7	176.86 (17)	O5—S2—C15—S2'	−51.4 (8)
N3—C7—C8—N2	177.5 (2)	C14—S2—C15—S2'	59.1 (8)
C11—C7—C8—N2	−1.3 (3)		

Symmetry codes: (i) −*x*+1, *y*+1/2, −*z*+1/2; (ii) −*x*+1, *y*−1/2, −*z*+1/2.

### Hydrogen-bond geometry (Å, º)

**Table:**

*D*—H···*A*	*D*—H	H···*A*	*D*···*A*	*D*—H···*A*
N3—H3···O3^iii^	0.88	2.17	3.042 (3)	173
C1—H1···O1	0.95	2.55	3.306 (4)	136
C5—H5···O2^iv^	0.95	2.32	3.151 (3)	145
C6—H6*A*···O3^v^	0.99	2.42	3.405 (4)	175
C8—H8···O3^v^	0.95	2.55	3.480 (4)	168
C10—H10···O4^vi^	0.95	2.44	3.309 (4)	152
C12—H12*A*···O5	0.98	2.43	3.377 (4)	161
C12—H12*B*···O5^vii^	0.98	2.55	3.478 (4)	159
C12—H12*C*···O3^viii^	0.98	2.35	3.270 (4)	156
C13—H13*A*···O1	0.98	2.53	3.292 (4)	134
C15—H15*C*···O2^iii^	0.98	2.59	3.470 (6)	149

Symmetry codes: (iii) *x*, *y*, *z*+1; (iv) −*x*+1, −*y*+1, −*z*; (v) *x*, −*y*+1/2, *z*+1/2; (vi) −*x*+1, −*y*+1, −*z*+1; (vii) *x*, −*y*+3/2, *z*−1/2; (viii) −*x*+2, −*y*+1, −*z*.
